# Renal Protection with SGLT2 Inhibitors: Effects in Acute and Chronic Kidney Disease

**DOI:** 10.1007/s11892-021-01442-z

**Published:** 2022-02-03

**Authors:** Clifford J. Bailey, Caroline Day, Srikanth Bellary

**Affiliations:** Life and Health Sciences, Aston University, Birmingham, B4 7ET UK

**Keywords:** Sodium-glucose co-transporter-2 (SGLT2) inhibitors, Diabetic kidney disease, Chronic kidney disease, Albuminuria, Acute kidney disease

## Abstract

**Purpose of Review:**

This review offers a critical narrative evaluation of emerging evidence that sodium-glucose co-transporter-2 (SGLT2) inhibitors exert nephroprotective effects in people with type 2 diabetes.

**Recent Findings:**

The SGLT2 inhibitor class of glucose-lowering agents has recently shown beneficial effects to reduce the onset and progression of renal complications in people with and without diabetes. Randomised clinical trials and ‘real world’ observational studies, mostly involving type 2 diabetes patients, have noted that use of an SGLT2 inhibitor can slow the decline in glomerular filtration rate (GFR), reduce the onset of microalbuminuria and slow or reverse the progression of proteinuria.

**Summary:**

The nephroprotective effects of SGLT2 inhibitors are class effects observed with each of the approved agents in people with a normal or impaired GFR. These effects are also observed in non-diabetic, lean and normotensive individuals suggesting that the mechanisms extend beyond the glucose-lowering, weight-lowering and blood pressure-lowering effects that accompany their glucosuric action in diabetes patients. A key mechanism is tubuloglomerular feedback in which SGLT2 inhibitors cause more sodium to pass along the nephron: the sodium is sensed by macula cells which act via adenosine to constrict afferent glomerular arterioles, thereby protecting glomeruli by reducing intraglomerular pressure. Other effects of SGLT2 inhibitors improve tubular oxygenation and metabolism and reduce renal inflammation and fibrosis. SGLT2 inhibitors have not increased the risk of urinary tract infections or the risk of acute kidney injury. However, introduction of an SGLT2 inhibitor in patients with a very low GFR is not encouraged due to an initial dip in GFR, and it is prudent to discontinue therapy if there is an acute renal event, hypovolaemia or hypotension.

## Introduction

Sodium-glucose co-transporter-2 (SGLT2) inhibitors are glucose-lowering agents that eliminate excess glucose through a glucosuric effect by reducing glucose reabsorption from the renal filtrate [1, 2•]. Since the introduction of the first SGLT2 inhibitor in 2012, the class has grown to include canagliflozin, dapagliflozin, empagliflozin and ertugliflozin in Europe and the Americas, with additional members of the class becoming established in other regions (Table 1). Although designed to reduce hyperglycaemia and assist body weight control in type 2 diabetes, further therapeutic opportunities are now recognised for SGLT2 inhibitors to address the cardio-renal complications and comorbidities of type 2 diabetes.

**Table 1:** SGLT2 inhibitors

Agent	Company	Brand	Dose mg/day	SGLT2 IC_50_ nmol/l	SGLT1 IC_50_ nmol/l
Dapagliflozin	AstraZeneca*	*Farxiga*	5, 10	1.2	1400
Canagliflozin	Janssen, Napp	*Invokana*	100, 300	2.7	710
Empagliflozin	Boehringer Ingelheim, Eli Lilly	*Jardiance*	10, 25	3.1	8300
Ertugliflozin	Merck Sharp & Dohme, Pfizer	*Steglatro*	5, 15	0.9	1960
Sotagliflozin	Lexicon	*Zynquista*	200	1.8	36
Ipragliflozin	Astellas	*Suglat*	25, 50	7.4	1875
Luseogliflozin	Taisho	*Lusefi*	2.5, 5	2.3	3990
Tofogliflozin	Chugai, Kowa	*Apleway*	20, 40	2.9	8444

This list of SGLT inhibitors includes the main members of the class approved for routine clinical use in the management of diabetes. Indications vary between regions and prescribers should consult their local product label. IC50 values are approximate. For comparison, phlorizin has an IC50 for SGLT1 of ~ 210 nmol/L and IC50 for SGLT2 of ~ 35 nmol/L

^*^Dapagliflozin was initially developed by Bristol Myers Squibb and is marketed in Europe as Forxiga

Initial concerns about SGLT2 inhibitors focussed on possible detrimental effects on the renal system, particularly to increase genito-urinary infections, compromise bladder health and aggravate acute kidney injury [3]. It was also noted with apprehension that administration of an SGLT2 inhibitor caused a temporary dip in glomerular filtration rate and caused persistent reductions in plasma volume and blood pressure. However, observations during cardiovascular outcome trials and ‘real world’ studies have identified potentially advantageous effects of SGLT2 inhibitors to reduce the risk of onset and progression of several cardiovascular conditions and to preserve kidney function.

This narrative review, which is based on an extensive literature review (Box 1), offers a critical appraisal of emerging evidence for the nephroprotective properties of SGLT2 inhibitors.

Box 1 Literature search strategy and selection criteria. MEDLINE, PubMed, and Google Scholar were searched for articles published between January 2010 and March 2021 using the terms ‘sodium glucose transporter inhibitor’, ‘SGLT2 inhibitor’ and the generic names of individual SGLT2 inhibitors in combination with the term ‘kidney disease’, ‘acute kidney injury’, ‘chronic kidney disease’, ’renal function’, ‘diabetes’ and ‘type 2 diabetes’. Studies were selected if they were conducted in human populations and/or described clinically relevant mechanisms, published in English and provided cogent information. Case reports, editorials, guidelines and preclinical studies were included when they offered information or interpretations not available in other sources.

## SGLT2 Inhibition

The development of SGLT2 inhibitors can be traced from the nineteenth-century observations that the glucoside phlorizin caused glucosuria [4]. Preclinical studies in the 1980s showed that phlorizin treatment could control hyperglycaemia in partially pancreatectomised rats, but clinical application awaited synthetic analogues that evaded intestinal glucosidase degradation and offered improved potency and selectivity to inhibit SGLT2 rather than SGLT1 [5, 6].

**Fig. 1:**
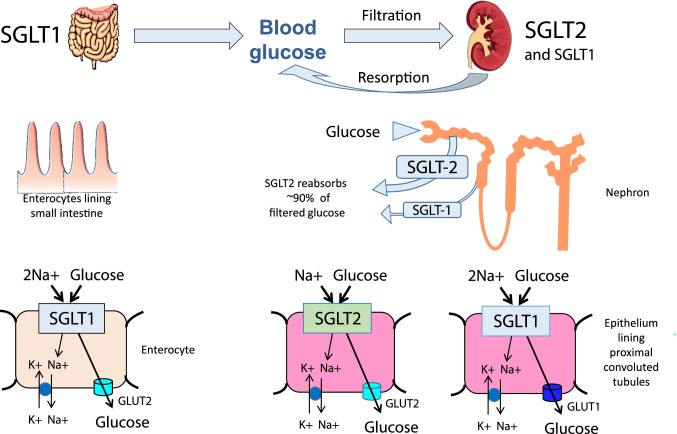
Key sites of action of sodium-glucose co-transporter (SGLT) inhibitors. SGLT2 (encoded by the solute carrier gene *slc5a2*) is expressed almost entirely in the luminal membrane of epithelial cells lining the first and second segments of the proximal tubules. It is a high capacity co-transporter acting with a sodium-glucose stoichiometry of 1:1 to mediate the reabsorption of most of the filtered glucose. SGLT1 (encoded by *slc5a1*) is expressed in the luminal membrane of cells lining the third (straight) segment of the proximal tubules. It acts with a sodium-glucose stoichiometry of 2:1 and has a lower capacity but higher affinity than SGLT2 to retrieve low concentrations of glucose remaining in the tubule. SGLT1 is expressed widely and occurs most abundantly in the apical membranes of enterocytes in the small intestine where it mediates glucose uptake from the intestinal lumen. Both transporters are secondary active symporters that depend on the sodium gradient created by Na + -K + -ATPase pumps in the basolateral membranes which lower the intracellular sodium concentration. Glucose that is taken up by sodium-glucose co-transporters into proximal tubule cells and enterocytes is eliminated across the basolateral membranes and into the interstitium via facilitative glucose transporters (e.g. GLUT1 and GLUT2)

SGLT2 is found almost exclusively in the luminal membranes of epithelial cells lining the first and second segments of the proximal tubules, where it mediates reabsorption of most (typically ≥ 90%) of filtered glucose (Fig. 1). SGLT1 in the luminal membranes of cells lining the third (straight) segment of the proximal tubules mediates reabsorption of low concentrations of glucose remaining in the tubule. SGLT1 is most abundant in the apical membranes of enterocytes where it mediates glucose uptake from the intestinal lumen. To avoid interference with the intestinal absorption of glucose, high selectivity for inhibition of SGLT2 has generally been preferred (Table 1). However, canagliflozin exerts some suppression of SGLT1, and sotagliflozin is an SGLT1/2 inhibitor: both of these agents can delay the intestinal absorption of glucose before being absorbed or degraded, which assists prandial glycaemic control. The amounts of these agents that are absorbed and exposed to the kidney, while inhibiting SGLT2, are insufficient to have any substantive inhibitory effect on SGLT1 in the proximal tubules [7, 8].

The hyperglycaemia of diabetes means that greater than normal amounts of glucose are filtered from the glomeruli into the proximal tubules, and increased amounts are reabsorbed, associated with a compensatory upregulation of SGLT2 and SGLT1 expression [9]. Nevertheless, the renal threshold for glucose is often breached, and the glucosuria is enhanced by SGLT2 inhibitors which act by reversible competitive inhibition without being transported themselves [2•]. They bind to the co-transporters at the luminal surface with greater affinity than glucose and with a residence time of several minutes. Thus, a trivial (therapeutic) concentration of an SGLT inhibitor in the filtrate can prevent the reabsorption of a large (up to about 100 g/day) amount of filtered glucose. Although SGLT2 inhibitors lower the renal threshold for glucose, their glucosuric effect is self-limiting such that they do not incur the risk of clinically significant hypoglycaemia. This is because, as the inhibitor increases glucosuria, this lowers blood glucose so that less glucose is filtered, and sufficient active (uninhibited) transporters can then reabsorb (almost) all of this lesser amount of glucose, which prevents blood glucose declining below euglycaemia.

## Glucose Lowering and Weight Lowering

The activity of SGLT2 inhibitors is independent of insulin status, i.e. undiminished by insulin resistance or absolute insulin lack, enabling glucose lowering in type 2 and type 1 diabetes. Dependency on the extent of hyperglycaemia means that glucose-lowering efficacy is greater in individuals with higher blood glucose concentrations and especially useful in reducing prandial glucose excursions. Meta-analyses of the reduction in HbA1c with SGLT2 inhibitors in type 2 diabetes have consistently noted reductions of about 0.5 to 1% (6–11 mmol/mol) from a baseline of around 8% (64 mmol/mol) [10–12]. Because SGLT2 inhibitors have a different mechanism of action to other glucose-lowering agents, they can be used in combination with other agents including insulin and can often reduce the amount of insulin required in type 2 and type 1 diabetes [13, 14]. However, SGLT2 inhibitors cannot replace the need for sufficient insulin to sustain fundamental metabolic requirements. Over reduction of (or delay in starting) insulin is usually the reason for atypical (euglycaemic) diabetic ketoacidosis (DKA) in which DKA occurs without hyperglycaemia and sometimes reveals that a diagnosis of type 2 diabetes is in fact type 1 [15].

In clinical trials with type 2 diabetes patients, the weight reducing effect of SGLT2 inhibitors has typically been around 3 kg, levelling out by 6–12 months, although ‘real world’ observational studies have often noted reductions > 6 kg that continue beyond a year. Declining weight is generally attributed to calorie loss through glucosuria reducing adipose mass, although reduced plasma volume may also contribute [16, 17].

## Cardiovascular Effects

Beyond glucose lowering and weight lowering, SGLT2 inhibitors have consistently reduced blood pressure (systolic by 3–5 mmHg and diastolic by 2–3 mmHg) during clinical trials without causing hypotension [18]. SGLT2 inhibitors have also consistently reduced the risk of new heart failure and worsening of existing heart failure during clinical trials (Table 2). The benefit is evident within a few weeks of starting an SGLT2 inhibitor, occurs in people with and without diabetes and is independent of the extent of effects on glucose, weight or blood pressure [19–21]. The improved prognosis for heart failure is also independent of age and is not significantly affected by the presence of CKD, albuminuria or concomitant use of antihypertensive therapies. Studies in which ejection fraction was quantified have mostly involved patients with reduced ejection fraction (HFrEF), but there is emerging evidence that SGLT2 inhibitors can also benefit those with preserved and mid-range ejection fraction. [22–25]. Measures of atherosclerotic cardiovascular disease (cardiovascular deaths, non-fatal myocardial infarction and stroke) have also been reduced in some studies with SGLT2 inhibitors: these are reviewed in detail elsewhere in the context of the reciprocating interrelationships of heart and kidney [19–21, 26, 27].

**Table 2:** Large randomised controlled cardiovascular outcome trials (CVOTs) in which renal events were measured during treatment of type 2 diabetes patients with an SGLT2 inhibitor. *BMI* body mass index, *CVD* cardiovascular disease, *eGFR* estimated glomerular filtration rate, *GLD* glucose-lowering drug, *MACE* major adverse cardiovascular event (cardiovascular death, non-fatal myocardial infarction or stroke), *MI* myocardial infarction, *UACR* urine albumin-creatinine ratio. Values for MACE, CV death, MI, stroke, heart failure, all deaths and renal composite are hazard ratio with 95% confidence intervals. *Renal composites varied between trials: EMPA-REG OUTCOMES, doubling of serum creatinine, eGFR ≤ 45 ml/min/1.73 m^2^, start renal replacement, renal death; CANVAS PROGRAM, > 40% decrease in eGFR, start renal replacement, renal death: DECLARE, > 40% decrease in eGFR, end stage kidney disease, renal or CV death; VERTIS, doubling of serum creatinine, start renal replacement, renal death; CREDENCE, double serum creatinine, end-stage kidney disease, renal death or CV death; SCORED, sustained (> 30 days) decrease of ≥ 50% in eGFR, dialysis and renal transplantation or sustained (> 30 days) eGFR of < 15 ml/min/1.73 m^2^. **Decline in long-term rate of eGFR

	Patients with type 2 diabetes					
	Cardiovascular outcome studies				Renal impairment studies	
Trial →	EMPA-REG	CANVAS (Program)	DECLARE	VERTIS	CREDENCE	SCORED
Agent	Empagliflozin	Canagliflozin	Dapagliflozin	Ertugliflozin	Canagliflozin renal	Sotagliflozin renal
Follow-up (median years) Date of trial end	3.1 yr 2015	2.4 yr 2017	4.5 2018	3.0 2019	2.6 2018	1.3 2020
*n*	7,020	10,142	17,160	8,246	4,401	10,584
Age (yr)	63	63.3	63.8	64.4	63.0	69
BMI (kg/m^2^)	30.6	32.0	32.1	31.9	31.3	31.8
HbA1c (%)	8.1	8.2	8.3	8.2	8.3	8.3
Diabetes duration	57% > 10y	13.5y	11.8y	13.0y	15.8y	-
Insulin ± GLD (%)	48	50	41	46.5	65.5	64
Prior CVD (%)	100	65	41	100	50	50
Heart failure (%)	12	11	9.9	23.4	14.8	31
MACE	**0.86** 0,74, 0.99	**0.86** 0,75, 0.97	**0.86** 0,74, 0.99	**0.97** 0,85, 1.11	**0.80** 0.67, 0.95	**0.77** 0.65, 0.91
CV death	**0.62** 0.49, 0.77	**0.87** 0,72, 1.06	**0.62** 0.49, 0.77	**0.92** 0.77, 1.11	0.78* 0.61, 1.00	**0.90** 0.73, 1.12
Non-fatal MI	**0.87** 0.70, 1.09	**0.85** 0,69, 1.05	**0.87** 0.70, 1.09	**1.04** 0.86, 1.27	**0.81** 0.59, 1.10	**0.68** 0.52, 0.89
Non-fatal stroke	**1.24** 0.92, 1.67	**0.90** 0,71, 1.15	**1.24** 0.92, 1.67	**1.00** 0.76, 1.32	**0.80** 0.56, 1.15	**0.66** 0.48, 0.91
Heart failure hospitalisation	**0.65** 0.50, 0.85	**0.67** 0,52, 0.87	**0.65** 0.50, 0.85	**0.70** 0.54, 0.90	**0.61** 0.47, 0.80	**0.67** 0.55, 0.82
All death	**0.68** 0.57, 0.82	**0.87** 0,74, 1.01	0.68* 0.57, 0.82	**0.93** 0.80, 1.08	**0.83** 0.68, 1.02	**0.99** 0.83, 1.18
eGFR range and mean eGFR (ml/min/1.73 m^2^)	> 30 74.0	> 30 76.5	> 60 85.2	> 30 76.1	30–90 56.2	25–60 44.5
Albuminuria UACR mg/g	17	12	13	19	927	74
Renal composite*	**0.54** 0.70, 0.75	**0.60** 0.47, 0.77	**0.53** 0.43, 0.66	**0.81** 0.63, 1.04	**0.70** 0.59, 0.82	**0.71** 0.46, 1.08
Decreased eGFR decline**	Yes	Yes	Yes	Yes	Yes	Yes
Decreased albuminuria	Yes	Yes	Yes	Yes	Yes	Yes

## Diabetic Kidney Disease

All types of diabetes are associated with increased risk of impaired kidney function (referred to as diabetic nephropathy or diabetic kidney disease (DKD)). This is typically recognised by a progressive chronic kidney disease (CKD) with an estimated glomerular filtration rate (eGFR) < 60 ml/min/1.73m^2^ that can be attributed to diabetes [28]. The condition may be accompanied by micro- (UACR 30–300 mg/g) or macro- (> 300 mg/g) albuminuria, often with an underlying glomerulopathy of thickened capillary basement membranes, diffuse mesangial sclerosis and nodular sclerosis. The normal age-related rate of decline in eGFR (~ 1 ml/min/1.73m^2^ per year when eGFR > 60 ml/min/1.73m^2^) is typically doubled in type 2 diabetes with CKD and may exceed 3 ml/min/1.73m^2^ per year in individuals with macroalbuminuria [29]. Observational studies suggest that 20–40% of people with type 2 diabetes incur an eGFR < 60 ml/min/1.73m^2^, mostly amongst older patients and those with poor glycaemic control. Type 2 diabetes is also a major cause of end stage kidney disease (ESKD) requiring renal replacement therapy [30–32].

Conventional treatments (mostly stringent control of blood pressure with ACE inhibitors or ARBs and intensive blood glucose control) reduce progression of DKD, but they have not been able to stop disease progression [33•, 34]. An emerging wealth of evidence now suggests that SGLT2 inhibitors can protect against the onset of DKD and slow disease progression independently of and additively to blockade of the renin–angiotensin–aldosterone system (RAAS).

## Kidney Disease and Use of SGLT2 Inhibitors

Because a reduction in GFR reduces the amount of glucose delivered into the proximal tubules, the glucosuric efficacy (and thereby antihyperglycaemic efficacy) of SGLT2 inhibitors declines approximately in line with a decline in GFR. In consequence, the product labels for SGLT2 inhibitors define GFR values below which it is recommended to not initiate or continue treatment [35–38]. With increasing appreciation that SGLT2 inhibitors do not jeopardise renal safety and offer cardio-renal benefits, the indications and permitted eGFR ranges have been expanded (Table 3) and vary between countries.

**Table 3:** Prescribing information for SGLT2 inhibitors available in the USA*

	Product label in USA	
	Indications	Renal impairment
Canagliflozin	Adjunct to diet and exercise to improve glycaemic control in adults with T2DM	Dose limited to 100 mg/day in patients with eGFR of 45—< 60 ml/min/1.73 m^2^ Do not initiate if eGFR < 45 ml/min/1.73 m^2^ Discontinue when eGFR persistently < 45 ml/min/1.73 m^2^
Dapagliflozin	Adjunct to diet and exercise to improve glycaemic control. And reduce the risk of hospitalisation for heart failure in adults with T2DM and established CVD or multiple CV risk factors To reduce the risk of CV death and hHF in adults with HFrEF (NYHA class II–IV)	No dose adjustment if eGFR ≥ 45 ml/min/1.73 m^2^ Not recommended for glycaemic control if eGFR 30- < 45 ml/min/1.73 m^2^ but can be used without dose adjustment to reduce risk of CV death and hHF in patients with HFrEF, with or without T2DM
Empagliflozin	Adjunct to diet and exercise to improve glycaemic control in adults with T2DM To reduce the risk of CV death in adults with T2DM and established CVD	Do not initiate if eGFR < 45 ml/min/1.73 m^2^ No dose adjustment if eGFR ≥ 45 ml/min/1.73 m^2^ Discontinue if eGFR persistently < 45 ml/min/1.73 m^2^
Ertugliflozin	Adjunct to diet and exercise to improve glycaemic control in adults with T2DM	Contraindicated if eGFR < 30 ml/min/1.73 m^2^ Initiation not recommended if eGFR 30—< 60 mL/min/1.73 m^2^ Continued use not recommended if eGFR persistently 30—< 60 ml/minute/1.73 m^2^ No dose adjustment needed in mild renal impairment

Product labels for the USA accessed 2 July 2021. Labels vary between countries. *CV* cardiovascular, *CVD* cardiovascular disease, *eGFR* estimated glomerular filtration rate by MDRD or CKD-EPI equations, *HF* heart failure, *hHF* hospitalisation for heart failure, *HFrEF* heart failure with reduced ejection fraction, *T2DM* type 2 diabetes mellitus

## Renal Endpoints

**Fig. 2:**
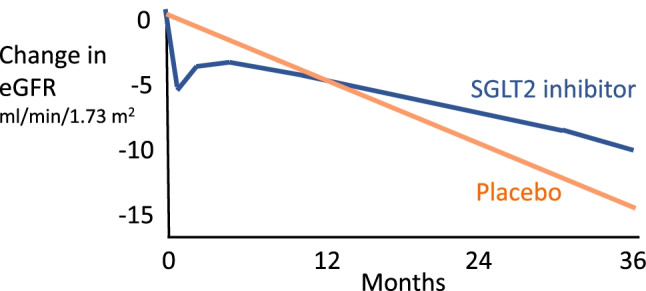
Illustration to show the typical changes in estimated glomerular filtration rate (eGFR) following the introduction and long-term use of SGLT2 inhibitors in people with type 2 diabetes. The initial dip in eGFR is about 5 ml/min/1.73 m^2^, reaches a nadir within 1–2 weeks and slowly returns towards pretreatment values over the next 3–9 months. Thereafter the rate of decline in eGFR is slower than in individuals who are not treated with an SGLT2 inhibitor. The illustration is loosely based on data from the EMPA-REG, CREDENCE and DAPA-CKD trials

Recognition that SGLT2 inhibitors could alter the course of diabetic kidney disease was initially clouded by short-term observations focussed on the initial dip in eGFR. This dip is typically about 5 ml/min/1.73m^2^, reaching a nadir within 1–2 weeks and slowly returning towards pretreatment values over the next 3–9 months (Fig. 2). However, evidence from long-term trials in type 2 diabetes, notably the post-marketing cardiovascular outcome trials (CVOTs) described below, indicated that eGFR subsequently declined at a slower rate with use of an SGLT2 inhibitor than in placebo-treated patients and that albuminuria was less severe.

Prespecified secondary endpoints in the CVOTs assessed various individual and composite measures of renal function that included progression of albuminuria (indicated by UACR), doubling of serum creatinine, decrease in eGFR (to either < 45 or < 60 ml/min/1.73m^2^), end-stage kidney disease, kidney-related death or renal replacement therapy (dialysis or transplantation). Because the composites and the patient populations differed between the trials, direct comparisons are necessarily cautious. However, each of the composites that included a measure of the rate of decline in eGFR noted a significant benefit of treatment with an SGLT2 inhibitor: for example, a decrease in adverse events by ≥ 30% (Table 2). Also, the individual renal parameters assessed in the CVOTs showed either significant reductions or non-significant numerical reductions in the occurrence of adverse renal events, bearing in mind that the studies were not powered for renal events.

## Large Randomised Trials in Type 2 Diabetes

In the EMPA-REG OUTCOME trial, the renal composite (doubling of serum creatinine, eGFR ≤ 45 ml/min/1.73 m^2^, initiation of renal replacement therapy or death from kidney disease) was reduced by 46% in the groups receiving empagliflozin [39, 40]. After the initial dip in eGFR, there was a slight annual decline in eGFR with use of empagliflozin (0.19 ± 0.11 ml/min/1.73 m^2^/year; mean ± standard error) compared with a more rapid decline in the placebo group (1.67 ± 0.13 mL/min/1.73 m^2^/year). Progression to macroalbuminuria (UACR > 300 mg/g) was reduced by 38% with use of empagliflozin, and there were also significant reductions in the number of patients with a doubling of serum creatinine, decline in eGFR to ≤ 45 ml/min/1.73 m^2^, and starting renal replacement therapy.

The DECLARE-TIMI 58 trial found that use of dapagliflozin was associated with a 47% reduction in a renal composite of a sustained decrease of eGFR by ≥ 40% to < 60 ml/min/1.73 m^2^, new ESKD or death from a renal cause [41]. The decline in eGFR (by ≥ 40% to < 60 ml/min/1.73 m^2^) was 46% less with dapagliflozin, and there were also significant reductions in ESKD and renal death. Additionally, dapagliflozin decreased new-onset albuminuria by 21% and new-onset macroalbuminuria by 46% [42].

Combined analysis of the CANVAS and CANVAS-R trials (CANVAS program) noted that use of canagliflozin reduced by 40% a renal composite of sustained (≥ 2 consecutive measures) reduction (by ≥ 40%) in eGFR, need for renal replacement therapy or death from renal causes [43]. Progression of albuminuria (change from normal to micro- or micro- to macro-albuminuria or ≥ 30% increase in micro-albuminuria) was reduced with canagliflozin by 27%, and many of the patients receiving canagliflozin showed a reduction of micro- or macro-albuminuria.

In the VERTIS CV trial, the renal composite, which did not include a measure of eGFR (doubling of the serum creatinine, starting renal replacement therapy or death from a renal cause), was numerically reduced by 19% (not statistically significantly) with use of ertugliflozin [44]. However, separate analysis of a renal composite comprising a sustained ≥ 40% reduction in eGFR, renal dialysis/transplant or renal death noted a 34% reduction with ertugliflozin, and by 5 years, the decline in eGFR was significantly less (by 2.6 ml/min/1.73 m^2^) than with placebo [45]. Also, by 5 years, ertugliflozin reduced progression from normo- to microalbuminuria by 21% and increased regression from macro- to micro- and from macro- or micro- to normoalbuminuria by 23%.

Several meta-analyses of the renal data from the above CVOTs and from other studies have confirmed that SGLT2 inhibitors reduced a composite of worsening eGFR, ESKD or renal death by about 33% [46–48].

## Trials in People with Impaired Renal Status

Across the four CVOTs described above, beneficial effects of the SGLT2 inhibitor on the various renal parameters were evident (to a greater or lesser extent) irrespective of gender, ethnicity, age, weight, duration or severity of diabetes; presence or absence of cardiovascular disease; and baseline eGFR or baseline albuminuria. However, in these studies, few patients had advanced CKD (e.g. eGFR < 45 ml/min/1.73 m^2^) or advanced macroalbuminuria. This was addressed in the CREDENCE study in which type 2 diabetes patients were recruited with an eGFR range of 30–90 ml/min/1.73 m^2^, macroalbuminuria (UACR > 300 to < 5,000 mg/g) and RAAS blockade [49].

In CREDENCE, 60% of patients had an eGFR of < 60 ml/min/1.73 m^2^, and 30% had an eGFR of < 45 mL/min/1.73 m^2^ (mean baseline eGFR of 56 ml/min/1.73 m^2^), while 88% had a UACR > 300–5000 mg/g (median UACR 927 mg/g). The renal composite (doubling of serum creatinine, ESKD, renal death or CV death) was 30% lower with use of canagliflozin, and there was a significant reduction in the rate of decline in eGFR for those receiving canagliflozin compared to placebo (–1.85 ± 0.13 versus –4.59 ± 0.14 ml/min/1.73 m^2^/year). If these different rates of decline in eGFR were continued for such a population (age 63, eGFR 56 ml/min/1.73 m^2^), it is calculated to take more than 10 years longer for the canagliflozin patients to progress into ESKD [49]. Indeed, in CREDENCE there were 32% fewer cases of ESKD (eGFR of < 15 ml/min/1.73 m^2^ and/or renal replacement) and 34% fewer renal deaths with use of canagliflozin. Also, canagliflozin lowered UACR by 31% at 6 months and increased by 30% the number of patients with a reduction in UACR [50]. Of particular note, the effectiveness of the SGLT2 inhibitor to slow the decline in eGFR and reduce progression of albuminuria was similar for patients with a baseline eGFR > or < 45 ml/min/1.73 m^2^ and a UACR > or < 1,000 mg/g, and the SGLT2 inhibitor also slowed the decline in eGFR for patients with a baseline eGFR < 30 ml/min/1.73m^2^. The effectiveness of the SGLT2 inhibitor on these parameters was independent of glycaemic status, type of RAAS blockade and atherosclerotic cardiovascular disease, suggesting that the benefits of SGLT2 inhibitors on renal function can be gained irrespective of cardio-renal or metabolic status in type 2 diabetes.

Similar findings emerged from the SCORED trial in type 2 diabetes patients with CKD (eGFR 25–60 ml/min/1.73 m^2^). Treatment with the SGLT1/2 inhibitor sotagliflozin was associated with a 29% reduction in the renal composite of sustained (> 30 days) decrease of ≥ 50% in eGFR, dialysis, renal transplantation or sustained (> 30 days) eGFR of < 15 ml/min/1.73 m^2^ [52].

Because the CVOTs and similar studies in type 2 diabetes indicated that the cardio-renal benefits of SGLT2 inhibitors were not contingent on their glucose-lowering efficacy, studies were undertaken in populations that included people without diabetes (Table 4). The DAPA-CKD trial examined the effect of dapagliflozin in people with (67%) and without (33%) type 2 diabetes who had renal impairment (eGFR 25–75 ml/min/1.73 m^2^, mean 43.1 ml/min/1.73 m^2^; and UACR 200–5000 mg/g, median ~ 950 mg/g with 48.3% of patients having a UACR > 1000 mg/g) [51]. Standard care for all patients included RAAS blockade. Similar reductions in the renal composite endpoint (decline in eGFR > 50%, ESKD, renal death or CV death) were observed with use of dapagliflozin in those with (by 36%) and without (by 50%) diabetes. Dapagliflozin also reduced each of the component measures of the composite, and the findings were generally consistent for patients with an eGFR > or < 45 ml/min/1.73 m^2^ or UACR > or < 1,000 mg/g. The average annual decline in eGFR was also slower with dapagliflozin than placebo (–1.67 versus –3.59 ml/min/1.73 m^2^).

**Table 4:** Large randomised controlled trials in which renal events were measured in ‘mixed populations’ of individuals with and without type 2 diabetes who were treated with an SGLT2 inhibitor. *BMI* body mass index, *eGFR* estimated glomerular filtration rate, *HFrEF* heart failure with reduced ejection fraction, *UACR* urine albumin-creatinine ratio. Values for renal composite are hazard ratio with 95% confidence intervals. *Renal composites varied between trials: DAPA-CKD, ≥ 50% decrease in eGFR, end-stage renal disease or renal or CV death; DAPA-HF, ≥ 50% sustained (> 28 days) decrease in eGFR, end-stage renal disease or renal death; EMPEROR-Reduced, dialysis or renal transplantation or sustained reduction of eGFR by ≥ 40% or eGFR < 15 ml/min/1.73 m^2^ if baseline eGFR > 30 ml/min/1.73 m^2^ or eGFR < 10 ml/min/1.73 m^2^ if baseline eGFR < 30 ml/min/1.73 m^2^. **Decline in long-term rate of eGFR. ***This value is a composite of CV death or hospitalisation for heart failure

	Patients with and without type 2 diabetes		
Trial →	DAPA-CKD	DAPA-HF	EMPA- REDUCED
Agent	Dapagliflozin renal	Dapagliflozin HFrEF	Empagliflozin HFrEF
Follow-up (median years) Date of trial end	2.4 2020	1.6 2020	1.3 2020
*n*	4,304	4,744	3,730
Age (yr)	61.8	66.2	66.8
BMI (kg/min^2^)	29.4	28.2	27.9
With type 2 diabetes (%)	67	42	50
% diabetes patients on insulin	55.0	27.6	73.8
Heart failure HFrEF (%)	10.9	100	100
CV death	**0.81** 0.58 1.12	**0.82** 0.69, 0.98	**0.92** 0.75,1.12
Heart failure hospitalisation	0.71*** 0.55, 0.92	**0.70** 0.59, 0.83	**0.69** 0.59, 0.81
All death	**0.69** 0.53–0.88	**0.83** 0.71, 0.97	**0.92** 0.77,1.10
eGFR range and mean eGFR (ml/min/1.73 m^2^)	25–75 43.1	≥ 30 66.0	> 20 61.8
Renal composite*	**0.61** 0.53, 0.88	**0.71** 0.44, 1.16	**0.50** 0.32, 0.77
Decreased eGFR decline**	Yes	Yes	Yes

## Additional Evidence from Populations With and Without Diabetes

The DAPA-HF and EMPEROR-reduced trials (with dapagliflozin and empagliflozin respectively) involved patients with and without diabetes, all of whom had diagnosed heart failure with HFrEF [22, 23]. Although these trials were focused on CV events, they reported less deterioration of a renal composite with use of the SGLT2 inhibitor, particularly a slower rate of decline in eGFR for people with and without diabetes independent of baseline renal status (Table 4) [53, 54].

DAPA-HF included patients with an eGFR down to 30 ml/min/1.73 m^2^ (mean 66 ml/min/1.73 m^2^) and noted that the renal composite (≥ 50% sustained decline in eGFR, ESKD or renal death) was numerically lower (by 39%, not statistically significant) with use of dapagliflozin [53]. However, the rate of decline in eGFR was significantly slower with dapagliflozin than placebo (mean –1.09 versus –2.85 ml/min/1.73 m^2^/year).

The EMPEROR-reduced trial recruited people with eGFR down to 20 ml/min/1.73 m^2^ (mean 62 ml/min/1.73 m^2^) and noted that use of empagliflozin reduced a renal composite (sustained decline in eGFR, dialysis or renal transplantation) by 50% [54]. Also, the decline in eGFR was significantly slower with empagliflozin than placebo (mean –0.55 versus –2.28 ml/min/1.73 m^2^/year).

Several studies to assess the long-term effects of SGLT2 inhibition in non-diabetic individuals with renal impairment are now ongoing, notably the EMPA-KIDNEY trial (ClinicalTrials.gov NCT03594110; https://www.clinicaltrials.gov/ct2/show/NCT03594110) involving use of empagliflozin in people with an eGFR down to 20 ml/min/1.73 m^2^.

## Renal Mechanisms of SGLT2 Inhibitors

The clinical trials described above have consistently indicated that use of an SGLT2 inhibitor can provide renal protection through a decreased rate of decline in eGFR and reduced onset or progression of albuminuria. This has been seen in people with and without diabetes and appears to be independent of the stage of CKD, the extent of albuminuria, ethnicity, age, gender, reduction of body weight or presence of cardiovascular disease. Although reduced glucotoxicity associated with sustained improvements in glucose homeostasis lessens the risks and severity of renal complications in type 2 diabetes irrespective of the glucose-lowering medication [33•], the renal benefits offered by SGLT2 inhibitors appear to be somewhat greater and faster in onset than with intensified glucose lowering and show little association with their glycaemic effect. So what might be the mechanisms?

### Osmotic Diuresis, Natriuretic and Hypovolaemia

When starting an SGLT2 inhibitor, the initial glucosuria is associated with an osmotic diuresis and natriuresis. The osmotic diuresis may be up to 400 ml/day depending on the hyperglycaemia but recedes as the hyperglycaemia recedes. The natriuresis is modest and mostly temporary, with little or no lasting change to fractional sodium excretion or plasma electrolyte concentrations, possibly reflecting a compensatory upregulation of aldosterone [55–58]. Although these initial effects are likely to contribute to a reduction in plasma volume and a decrease in blood pressure, they do not correlate with the maintenance of lower blood pressure [59, 60]. While a lower blood pressure will undoubtedly contribute to renal protection, the benefits reported with use of SGLT2 inhibitors appear to be more consistent and greater than noted with diuretic therapy [61], possibly involving differences in fluid redistribution between the intra- and extracellular compartments with SGLT2 inhibitors and standard thiazide and loop diuretics [62].

### Tubuloglomerular Feedback

Inhibition of SGLT2 leaves a higher than normal concentration of sodium in the lumen of the proximal tubule. This sodium passes through the loop of Henle and is sensed by cells of the macula densa at the top of the ascending limb [63••, 64, 65]. Uptake of this sodium by the macula cells exceeds the capacity of their Na^+^-K^+^ ATPase in the basolateral membrane causing an increased intracellular sodium concentration. This creates an osmotic gradient which causes the cells to swell and leak ATP across the basolateral membrane. The ATP is converted to adenosine by an extracellular nucleotidase, and the adenosine binds to adenosine A1 receptors on vascular smooth muscle cells lining afferent glomerular arterioles. This alters calcium fluxes and causes vasoconstriction which reduces blood flow into the glomerulus, decreasing intraglomerular pressure and thereby offering a mechanism to preserve glomerular viability.

Adenosine-mediated effects on calcium fluxes could also reduce the secretion of renin from juxtaglomerular cells which is expected to reduce RAAS-mediated vasoconstriction. However, some studies have noted a small initial increase in plasma renin activity after initiation of SGLT2 inhibition, but no clear consistent long-term changes in RAAS activity or interactions with RAAS blockers have been established [63••, 66, 67]. Macula densa cells express SGLT1 which mediates glucose uptake linked to nitric oxide (NO) production [68]. The NO can cause local vasodilatation, and animal studies suggest that extra glucose in the tubule might increase NO production by the macula densa sufficiently to affect glomerular filtration [69].

### Tubular Oxygenation

Hyperglycaemia increases filtration of glucose which increases glucose reabsorption in the proximal tubule. This in turn increases oxygen utilisation and depletes the oxygen supply to distal regions of the tubule, especially the renal medulla [70, 71]. By reducing glucose reabsorption, SGLT2 inhibitors may improve oxygen availability, reduce reactive oxygen species and improve viability of the medulla. This is not easily reconciled with evidence that SGLT2 inhibitors increase erythropoietin production, which is usually associated with renal hypoxaemia [72], although it does help to account for an increase in haematocrit which in turn would assist oxygen supply. To explain the dilemma, it has been suggested that by altering the lipid-glucose balance of nutrient metabolism in kidney tissue, SGLT2 inhibition alters the production or signalling of hypoxia-inducible factors (HIFs). This could reduce HIF-1 activity and/or promote HIF-2α activity, favouring a decrease in pro-inflammatory and fibrotic factors while also increasing erythropoietin [73, 74].

### Tubular Energetics and Sodium-Hydrogen Exchange

The alteration in nutrient metabolism proposed to affect HIFs is related to the excess reabsorption of glucose in diabetes causing a shift from fatty acid oxidation to glycolysis for energy production in proximal tubular cells [73, 74]. This then causes cell damage and fibrosis through an accumulation of intracellular lipid, whereas SGLT2 inhibition reduces reliance on energy production from glucose, increases fatty acid utilisation and reduces lipotoxic cell damage [75].

SGLT2 inhibitors suppress the activity of the sodium-hydrogen exchanger-3 (NHE-3) which has the potential to influence renal sodium handling, acid–base and metabolic activities at least partly independently of the inhibition of glucose reabsorption [66, 76]. The impact of SGLT2 inhibition on renal protection through this mechanism is unclear.

### Inflammation and Fibrosis

Several preclinical and clinical studies have noted that treatment with an SGLT2 inhibitor is associated with reductions in circulating markers of inflammation and fibrosis, notably nuclear factor-κB (NFκB), interleukin 6 (IL-6), monocyte chemoattractant protein 1 (MCP-1), tumour necrosis factor receptor 1 (TNFR1), matrix metalloproteinase 7 and fibronectin-1 [77]. A potential pro-inflammatory mechanism operating in the kidney via altered nutrient metabolism is considered above. Additionally, hyperuricaemia is associated with increased renal interstitial fibrosis, and SGLT2 inhibitors reduce plasma uric acid, probably through increased renal urate elimination due to competition of extra glucose for the urate transporter GLUT9b [78]. Possibly related is the evidence that there is reduced nephrolithiasis with use of an SGLT2 inhibitor [79].

### Other Mechanisms

Several further mechanisms have been considered to assist in the renoprotective effect of SGLT2 inhibition. As illustrated by their ability to lower blood pressure without raising heart rate, SGLT2 inhibitors may reduce excess sympathetic activity [80]. Also, by reducing heart failure, SGLT2 inhibitors will reduce venous congestion and backpressure against the renal venous drainage.

## Cautions

Recommendations regarding the use of SGLT2 inhibitors in renal impairment are considered above (Table 3), and several additional cautions associated with the use of SGLT2 inhibitors are well recognised and can be managed as summarised in Table 5 [35–38]. Despite initial concerns, routine use of SGLT2 inhibitors has not increased urinary tract infections and has placated caution with regard to acute kidney injury (AKI)—elaborated below [81].

**Table 5:** Cautions associated with the use of SGLT2 inhibitors

Caution	Comment
Genito-urinary mycotic infections	Caution if history of frequent or severe prior infection Usually dealt with by hygiene advice and clotrimazole cream
Initial nocturia and orthostatic hypotension	Advice on care when getting up, especially at night
Hypovolaemia and dehydration	Advice to take in sufficient fluid especially in hot climates Awareness of relevant symptoms and ‘sick day’ rules
‘Atypical’ euglycaemic ketoacidosis	Mostly indicates under insulinisation because the glucosuria has lowered the plasma glucose, but there is insufficient insulin to prevent excess lipolysis. The resulting release of excess fatty acids gives rise to excess ketones. Avoid over-ambitious reductions of insulin dose. Consider if misdiagnosis of type 1 as type 2 diabetes
Urinary tract infections (UTI) and acute kidney injury (AKI)	Contrary to initial concerns, risk of UTI and AKI have been less common with use of an SGLT2 inhibitor
Risk of bone fracture	Unconfirmed with extensive routine use
Risk of lower limb amputation	Unconfirmed with extensive routine use, but vigilance suggested in patients with severe peripheral artery disease
Fournier’s gangrene	Very rare, association with SGLT2 inhibitors unclear
Interaction with antihypertensive medications	Dose adjustments to existing medication with a loop and/or thiazide diuretic or RAAS blocker may be required when starting an SGLT2 inhibitor to prevent volume depletion and orthostatic hypotension

*RAAS* renin–angiotensin–aldosterone system

## Acute Kidney Injury

AKI accounts for about 12% of hospital admissions in the USA and is reported to be more common and result in worse outcomes amongst people with diabetes [82–84]. Estimates of increased absolute risk of AKI with diabetes have varied considerably, probably due to the strong influence of diverse underlying pathologies that are manifest as low GFR, albuminuria, hypertension and co-existent CV disease, but the occurrence of AKI is generally more common in T1DM than T2DM. Given the alterations of renal function associated with the use of SGLT2 inhibitors, there has been ongoing caution regarding possible detrimental effects of these agents on the risk and prognosis of AKI. However, clinical trial data have consistently found no increase in risk of AKI or adverse outcomes of AKI with use of an SGLT2 inhibitor. Moreover, several meta-analyses of clinical trial data have concluded that use of an SGLT2 inhibitor in T2DM reduces the risk of AKI by 30–40%, and analyses of data from ‘real world’ observational studies have indicated reductions in risk of AKI by > 40% [81, 85, 86].

**Fig. 3:**
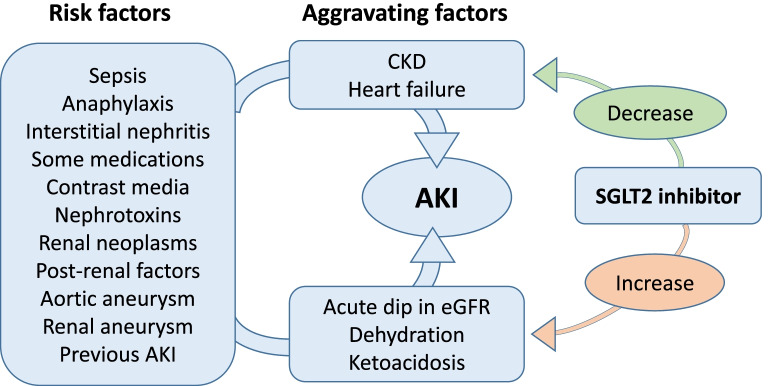
Schematic diagram to show the potential effects of SGLT2 inhibition on acute kidney injury (AKI). Use of an SGLT2 inhibitor is not a recognised risk for the occurrence of AKI, and available evidence indicates that SGLT2 inhibitors may be associated with a reduced occurrence of AKI. SGLT2 inhibitors may alter factors that ‘aggravate’ the severity of AKI. For example, SGLT2 inhibitors might improve the prognosis for people with AKI by decreasing the rate of decline of estimated glomerular filtration rate (eGFR) in people with chronic kidney disease (CKD) and by reducing the severity of heart failure. SGLT2 inhibitors might impair the prognosis for people with AKI if the SGLT2 inhibitor has been started recently and there is a drug-induced dip in eGFR and by dehydration or ketoacidosis

Many of the factors that can precipitate AKI appear to have little direct connection to the use of an SGLT2 inhibitor (such as sepsis, anaphylaxis, aortic aneurism or non-vascular post-renal factors). However, other precipitants of AKI could be associated with a potentially beneficial impact of SGLT2 inhibition (such as reduced CKD, heart failure and hyperglycaemia) or a potentially detrimental impact (such as hypovolaemia or ketoacidosis) (Fig. 3).

With regard to CKD, the ability of SGLT2 inhibitors to slow the age-related deterioration of GFR is consistent with a reduced risk and better prognosis of AKI [63••, 64]. Nevertheless, in individuals who already have a very low GFR, the initial dip in GFR that typically accompanies the introduction of an SGLT2 inhibitor could accentuate the risk of an acute adverse event [65]. Thus, product labels and guidelines do not recommend initiation of an SGLT2 inhibitor in routine clinical practice if eGFR is < 45 ml/min/1.73 m^2^ [35–38].

The reduced risk and severity of heart failure associated with use of an SGLT2 inhibitor, particularly HFrEF, provides a potentially important contribution to the reduced occurrence and better prognosis of AKI with this class of glucose-lowering agents [63••, 64]. Also, AKI with heart failure (‘cardio-renal syndrome’) may be associated with renal venous hypertension. This is sometimes responsive to diuretic therapy, and the diuretic effect of SGLT2 inhibition offers a further potential beneficial mechanism [87]. By lowering chronic hyperglycaemia, particularly in the efferent glomerular vessels supplying the renal medulla, SGLT2 inhibition is anticipated to reduce the risk of tubular damage from glucotoxicity and reactive oxygen species, thereby improving tubule viability which may help to mitigate the detrimental impact of AKI [88, 89]. The reductions of inflammatory infiltrate in the kidney interstitium and reduced tubulointerstitial fibrosis noted with SGLT2 inhibitor therapy in preclinical models suggests a further possible mechanism to reduce susceptibility to AKI [90, 91].

Significant hypovolaemia, which has been reported occasionally with SGLT2 inhibition, presents a potential risk for AKI. This adds caution to concomitant use of a standard diuretic and reinforces the reminder for patients to maintain adequate fluid intake. The atypical type of DKA noted with use of an SGLT2 inhibitor presents another risk for AKI and emphasises the need to ensure sufficient insulinisation. As noted earlier, product labels for SGLT2 inhibitors vary with regard to the recommended eGFR for starting and stopping these agents, and their use in ESRD is not currently supported. Thus, in patients presenting with AKI, use of an SGLT2 inhibitor should be stopped to avoid possible aggravation of low plasma volume, low blood pressure and low glomerular perfusion.

## Conclusion

Damage to the glomerulus often starts with the hyperfiltration that accompanies hyperinsulinaemic insulin resistance in prediabetes and early stages of type 2 diabetes. This sets in train the profibrotic changes, faster decline in GFR and functional disturbances of diabetic kidney disease. SGLT2 inhibitors exert a variety of effects on the kidney, directly and indirectly linked to reduced glucose reabsorption, providing acute and chronic nephro-protective effects that reduce progression and may partially reverse the characteristic markers of diabetic kidney disease.
